# A critical role for the NFkB pathway in multiple myeloma

**DOI:** 10.18632/oncotarget.109

**Published:** 2010-05-07

**Authors:** Yulia N. Demchenko, W. Michael Kuehl

**Affiliations:** ^1^*Genetics Branch, National Cancer Institute, Bethesda, MD, USA*

**Keywords:** Multiple myeloma, NFkB, mutations, cancer, chemotherapy, leukemia, drug resistance

## Abstract

NFkB transcription factors play a key role in the survival and proliferation of many kinds of B-cell tumors, including multiple myeloma (MM). It was shown that NFkB activation in MM tumors results mainly from extrinsic signaling by APRIL and BAFF ligands that stimulate receptors on normal plasma cells as well as on pre-malignant monoclonal gammopathy of undetermined significance (MGUS) and MM tumors. However, the mutations that occur during MM progression and that constitutively activate NFkB would be expected to decrease dependence of tumor cells on the bone marrow microenvironment. These mutations can activate the classical or alternative NFkB pathways selectively, but usually both pathways are activated in MM. Significantly, activation of either NFkB pathway leads to a similar response of MM cell lines. This frequent activation of the alternative pathway distinguishes MM from other B-cell tumors, which more frequently have mutations that are predicted to activate only the classical NFkB pathway. Given the strong dependence of MGUS and MM tumors on NFkB pathway activation, inhibition by a combination of targeting extrinsic signaling plus both NFkB pathways appears to be an attractive therapeutic approach in MM tumors.

## INTRODUCTION

Multiple myeloma (MM) is a post-germinal center tumor that has undergone extensive somatic hypermutation, antigen selection, and productive IgH switch recombination prior to homing to the bone marrow as a plasmablast that displays a long-lived plasma cell (PC) phenotype in this microenvironment. MM usually is preceded by non-IgM monoclonal gammopathy of undetermined significance (MGUS), a premalignant tumor that progresses to MM at an average rate of about 1% per year [1]. Both MGUS and MM, until terminal stages, are located mainly in the bone marrow.

There are several molecular subtypes of MGUS and MM, as defined by specific genetic and cytogenetic aberrations [2-4]. Early events distinguish two major categories of tumors that have a similar prevalence: hyperdiploid and non-hyperdiploid [5, 6]. Hyperdiploid tumors rarely have primary IgH translocations but usually have multiple trisomies involving eight odd-numbered chromosomes. By contrast, most non-hyperdiploid tumors have primary IgH translocations that usually occur as a result of errors in somatic hypermutation and IgH switch recombination in germinal center B-lymphocytes. The dysregulation of a Cyclin D gene is the unifying – perhaps initiating – event that is common to all MGUS and MM tumors. Significantly, MM tumors that are hyperdiploid or have a t(11;14) (CYCLIN D1) translocation have a better prognosis than tumors that have t(4;14)(MMSET&FGFR3) or MAF translocations.

The molecular basis for the progression of MGUS to MM is not understood. However, progression events that can occur in all of the different molecular subtypes of MM include: additional cytogenetic and epigenetic abnormalities, activating mutations of N- and K-RAS, AKT pathway mutations, NFkB pathway mutations, p53 deletion or mutation, RB1 or p18CDKN2c inactivation, and MYC rearrangements[2, 6-10].

In addition to an accumulation of genetic aberrations in MM cells, disease progression is mediated by evolving crosstalk between different cell types within the BM. MM cells can survive and proliferate in the BM microenvironment through their interactions with bone marrow stromal cells (BMSC) and extracellular matrix. By binding MM cells to BMSCs, adhesion molecules can activate intracellular signaling cascades in MM cells, and can increase stromal cell secretion of cytokines involved in tumor cell proliferation [11]. For example, IL6 production by BMSCs is induced by MM cells [12]. Some of the growth and survival factors produced in the BM microenvironment include: IL6, IGF, HGF, VEGF, osteopontin, SDF1, B cell– activating factor (BAFF), A proliferation-inducing ligand (APRIL). Moreover, adhesion of MM cells to BMSCs induces the release of different osteoclast activating factors by stromal and myeloma cells [13]. The crosstalk among the MM cells, osteoclasts, and stromal cells not only stimulates MM cell survival and growth, but also results in bone destruction by osteoclasts [14, 15].

Of all the different signaling pathways activated in MM cells by external stimulation, the NFkB pathway may be one of the most important. Some factors that are produced by BM cells, e.g., VEGF and IGF1, can indirectly activate the NFkB pathway in PC and MM cells [11, 16]. More importantly, it has been shown that BAFF and APRIL, which can directly activate the NFkB pathway, are two of the main survival factors for healthy PCs and MM cells [17, 18]. They share two receptors, TACI and BCMA, which activate the classical NFkB pathway, but BAFF also can activate the alternative pathway through the BAFF-R receptor.

Since myeloma tumors as well as many other B-cell neoplasms use NFkB to achieve survival, proliferation and resistance to anticancer drugs, inhibition of NFkB activation appears to be a very promising option for anti-cancer therapies.

### NFkB transcription factors and their signaling pathways

**Fig. 1:**
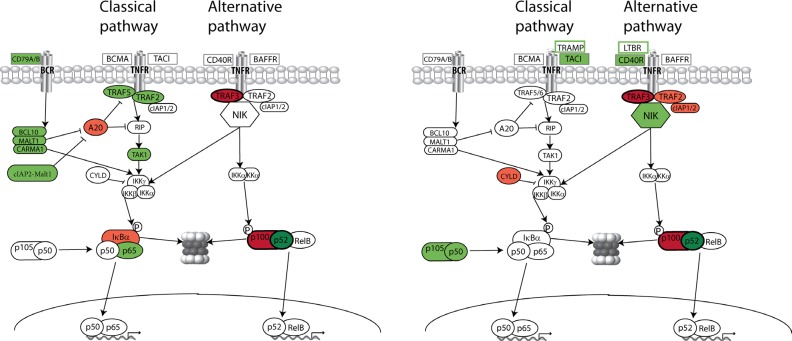
NFkB mutation in B-cells neoplasms **(A)** Frequent mutations in positive (green) and negative (red) regulators of NFkB pathway in mature B-cells neoplasms. **(B)** Frequent mutations in positive (green) and negative (red) regulators of NFkB pathway in multiple myeloma. Similar mutations for MM and other B-cell tumors are indicated with dark green and red colors. Components within green boxes are TNFR that are over-expressed in MMCL; but the significance of these two abnormalities remains to be clarified, i.e., FR4 MMCL has relatively low NFkB activity despite increased expression of LTBR [30]; it is not confirmed that markedly increased TRAMP expression is the cause of increased NFkB activity in the KMS-12BM and KMS-12PE MMCL.

The NFkB family of transcription factors is composed of NFKB1 (p50 and its precursor p105), NFKB2 (p52 and its precursor p100), RelA (p65), RelB and c-Rel [19, 20]. There are two general pathways of activation – classical (or canonical) and alternative (Figure 1). Many different stimuli, including external signaling through B cell receptors (BCR) and some tumor necrosis factor receptors (TNFR), activate the classical NFkB pathway [19]. Following stimulation through TNFR-associated factors (TRAF5, TRAF2), RIP and TAK1, IKKβ (which is part of an IKKα-IKKβ-IKKγ complex) is activated and phosphorylates the inhibitory subunits IkBα, IkBβ, and IkBε, leading to their proteasomal degradation. As a result, NFkB homodimers and heterodimers, comprised mainly of RelA, RelC, and p50, accumulate in the nucleus. Classical RelA:p50 heterodimers are predominantly regulated by IkBα. Several negative regulators, such as A20 (also known as TNFAIP3) and CYLD, are also important for the classical NFkB pathway. This pathway plays a major role in the control of immune response and inflammation, and is required to enhance the survival and proliferation of cells.

In the alternative pathway, NIK (NFkB-inducing kinase) activates IKKα, which phosphorylates NFKB2. This results in proteasomal removal of an inhibitory C-terminal IkBδ domain, generating the p52 subunit, which leads to accumulation of p52/RelB heterodimers in the nucleus. The alternative NFkB pathway, which is important in lymphoid development and B-cell maturation [21], is stimulated by a more restricted set of cytokines, including CD40L, LTαβ, BAFF, RANKL (receptor activator of NFkB ligand), and TWEAK (TNF-related weak inducer of apoptosis) [22-24]. Several recent reports indicate that the alternative NFkB signaling is regulated mainly through the control of NIK turnover, with TRAF3, TRAF2 and cIAP1/2 critically involved in this process [25-28].

### Importance of NFkB pathway for mature B-cells and PC, and their tumors

To investigate mechanisms of activation of NFkB in MM cells we created an NFkB index as a measure of NFkB activity [29]. We used the average RNA expression of eleven genes (nine of which are well known NFkB targets), that were down-regulated in MM cell lines (MMCLs) treated with MLN120b (IKKβ inhibitor) or transfected with antiNIK shRNA. The same 11 genes were up-regulated in MMCL transfected with IKKβ or NIK genes that activate, respectively, the classical and alternative NFkB pathways [30].

**Fig. 2:**
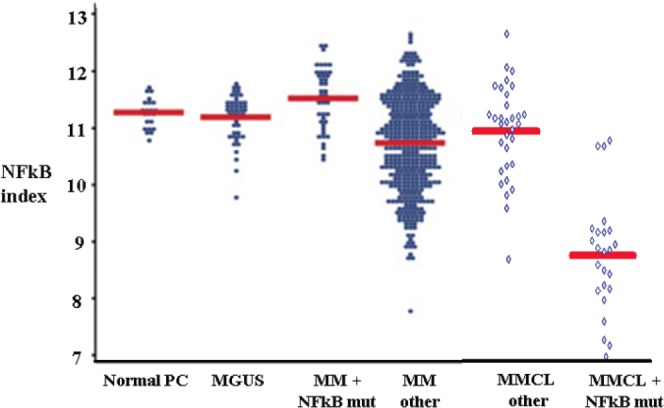
The levels of the NFkB activity in normal and malignant plasma cell types NFkB indices were calculated using the average expression of 11 NFkB target genes. The mean expression level is shown by the horizontal (red) line. (Figure was created by addition of new data to figure from Annunziata et al [29]).

**Fig. 3:**
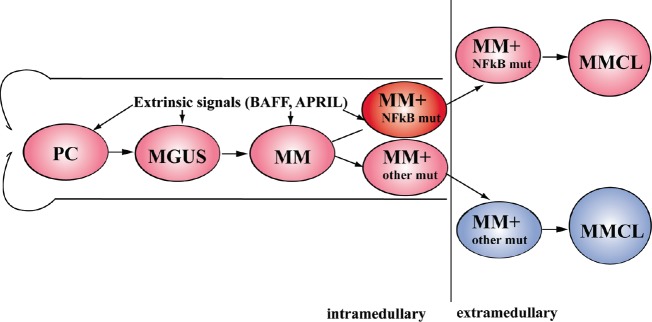
High level of the NFkB activity in normal and malignant PC results from extrinsic signals and/or NFkB mutations The colors correspond to the different levels of the NFkB activity – low activity (blue), high activity due to extrinsic or mutational activation (pink) and highest activity due to extrinsic plus mutational activation (red).

As we recently showed [29] (Fig.3D), various stages of normal human B cell differentiation have a different NFkB index. With the exception of germinal center B cells, the NFkB index was higher for mature B cells and B cells at later developmental stages compared to immature B cells and B cells at earlier developmental stages. The highest NFkB index was found in PC, consistent with their dependence on extrinsic BM signals that increase NFkB activity [31]. In addition, the level of NFkB activity was shown to be at a similarly high level in healthy and transformed PC, although MM tumors with mutations in the NFkB pathway had a somewhat higher NFKB index. In contrast, although MMCLs with mutations in the NFkB pathway also had a high NFKB index, MMCLs without apparent mutations in the NFkB pathway had a substantially lower NFkB index (Fig. 2) [29]. Together, these data suggest that NFkB activation results mainly from extrinsic TNFR signaling in healthy PC, and most MGUS and MM tumors. Mutations in the NFkB pathway in MM tumors presumably result in less dependence on – and in some cases independence from - extrinsic signals from the BM microenvironment.

Previously, it was shown that TACI-Fc, which acts as an antagonist to BAFF and APRIL by binding these factors and preventing them from interacting with TACI, BMCA, and BAFF.R receptors, causes a marked decrease in the number of normal murine BMPC. Strong dependence of MM cells on external activation of NFkB has also been demonstrated in experiments with Atacicept (formerly TACI-Ig), a recombinant fusion protein containing the extracellular, ligand-binding portion of the receptor TACI and the modified Fc portion of human IgG1. Inhibition of BAFF and APRIL using Atacicept in some MMCLs co-cultured with supporting osteoclasts or dendritic cells resulted in reduced myeloma cell growth and diminished clonogenic potential [32-34]. In addition, the first clinical study of Atacicept in MM patience demonstrated that about half of patients who completed initial treatment were progression-free after therapy [35].

It is notable that NFkB is important also in other kinds of normal mature B cells (perhaps with the exception of germinal center B cells), and especially in several B cell neoplasms. For instance, gene expression profiling separated Diffuse Large B Cell Lymphoma (DLBCL) into three gene expression subgroups: Germinal Center B cell-like (GCB), Primary Mediastinal B cell Lymphoma (PMBL) and Activated B Cell-like (ABC) [36-38]. These DLBCL subgroups arise from different stages of normal B cell differentiation. One significant difference among the DLBCL subgroups is the constitutive activity of the NF-κB pathway in ABC and PMBL but not GCB DLBCL, which is associated with a better outcome. It was found, that ABC and PMBL cells have high expression of known NFkB target genes when compared with GCB DLBCLs. Moreover, inhibition of the NFkB pathway using a dominant active form of IκBα, or a dominant negative form of the IKKβ subunit, was toxic to ABC DLBCL cell lines but not to GCB DLBCL cell lines [36].

### Mutations mostly activate the classical NFkB pathway in B-cell tumors

Since NFkB activity is high and critical for survival of healthy mature B-cells, and also is important in some kinds of B-cell tumors derived from mature B-cells, it is not surprising that mutations in the NFkB pathway are common in many kinds of B-cell neoplasms. Various kinds of NFkB pathway mutations have been found in Hodgkin's lymphoma (HL) and non-Hodgkin's lymphoma (NHL), including DLBCL, mucosa-associated lymphoid tissue lymphoma (MALT.L), follicular lymphoma (FL), and Waldenstrom's Macroglobulinemia (WM).

The BCR pathway is required for cell survival in normal B cells, and includes activation of the classical NFkB pathway. Classic HL, which is characterized by the presence of Hodgkin and Reed-Sternberg cells, are derived from the clonal expansion of a germinal centre B cell but often do not express the BCR and other B-cell markers [39]. Perhaps, the high prevalence of NFkB abnormalities in HL is able to compensate for survival mechanisms mediated in the absence of a BCR. In any case, in HL [40] and other B cell tumors several kinds of abnormalities have been found in the protein complex that transduces NFkB activating signals from the BCR. MALT1 was initially identified as an oncogenic protein commonly expressed in a subset of MALT.L. Early studies revealed that MALT1 binds to the BCL10 protein [41]. Further experimental evidence suggested that BCL10 and MALT1 cooperate with Carma1 (CARD11) [42] to form a trimeric protein complex (CBM), which is implicated in NFkB activation. This CBM complex can be recruited to the IKK complex, which leads to IKKβ phosphorylation [43]. Moreover, MALT1 was found to cleave human A20 (also called TNFAIP3) at Arg439, generating two protein fragments that did not inhibit NFkB activation [44]. Three recurrent translocations have been identified in MALT.L: the t(11;18)(q21;q21) *cIAP-MALT1* translocation, which is the most common; but also the t(1;14)(p22;q32) and t(14;18)(q32;q21) translocations, which place the Ig heavy chain enhancer upstream of the *BCL10* and *MALT1* genes, respectively, causing de-regulated expression of each protein (reviewed in [45]). Patients with the *cIAP2–MALT1* translocation have a poorer clinical prognosis than patients with other translocations [46-48]. This may be explained by results showing that *cIAP2– MALT1* fusions can more strongly activate the NFkB pathway in comparison to overexpression of either BCL10 or MALT1. Significantly, RNA interference screens have demonstrated that *CARD11*, BCL10 and MALT1, molecules are essential for NFkB activation and cell survival in ABC DLBCL cell lines [49]. Furthermore, it recently was shown that about 10% of ABC DLBCL have a mutant form of the *CARD11* BCR signaling adaptor, and that 18% have mutated the first ITAM tyrosine of *CD79B* (a proximal BCR subunit) [50].

One of the most frequent abnormalities, which were found in a wide range of B-cell neoplasms, is a loss of function of the A20 protein, a key negative regulator of the NFkB classical pathway. This negative regulator can be inactivated by somatic mutations or deletions in MALT.L (21.8%), HL of nodular sclerosis histology (33-44%), ABC DLBCL (24.3%), PMBL-DLBCL (36%) and, to a lesser extent, in FL, GCB DLBCL and WM [51-54]. It was shown, that in A20-deficient cells, re-expression of A20 leads to suppression of cell growth and NFkB activity [52].

Several other genetic alterations that contribute to activation of NFkB have been described. Inactivating mutations or deletions of IkBα have been identified in 10 % of HL [39, 55]. Furthermore, 20% of ABC DLBCL and a smaller fraction of GCB DLBCL carry somatic mutations in *TRAF2*, *TRAF5*, *TAK1* and *RANK* genes [54]. Amplification of *Rel* on chromosome 2p14-15 has been detected in HL (26%) and in a smaller proportion of PMBL-DLBCL, FL and MALT.L [56, 57]. This mutation is associated with high levels of nuclear c-Rel. Curiously, this amplification occurs also in 16% of GCB DLBCLs, but cells with this abnormality had largely cytoplasmic c-Rel [58] and do not express NFkB target genes at higher levels than those with a wild type *c-Rel* copy number [38].

Most genetic abnormalities in B-cell tumors result in activation of the classical NFkB pathway (Fig. 1A), with only two examples of mutations that would be predicted to activate the alternative NFkB pathway. The first example is structural alterations affecting the 3' portion of the *NFKB2* gene, which were found in some B-cell lymphoma [59, 60]. Although this is expected to specifically activate the alternative pathway, the mutations eliminate the carboxyterminal sequences, which inactivate the IkBδ activity that can be a significant inhibitor of the classical pathway. The second example is biallelic inactivation of the *TRAF*3 gene in 5% of WM [53], but also in B-cell NHL and chronic lymphocytic leukemia that have deletion del(14)(q24.1q32.33) [61]. As described below, inactivation of *TRAF*3 results in increased levels of NIK protein that activates the alternative NFkB pathway, but sometimes can also activate the classical NFkB pathway. It remains to be determined if activation of the classical NFkB pathway is the uniquely important biological consequence of the NFkB pathway mutations that have been found in B-cell tumors.

### Mutations can activate either but usually both NFkB pathways in MM

Mutations in eleven genes involved in regulation of the NFkB pathway have been identified in 42% of MMCL, and at least 17% of MM tumors (Fig. 1B) [29, 62]. Translocations or amplification was found to increase transcription of five positive regulators, which include *NIK* and *NFKB1* plus three TNFR (*CD40*, *TACI*, *LTBR*). In addition, there was one example of a translocation that deleted a negative regulatory region from *NIK* so that it was less susceptible to proteasomal degradation. In contrast, deletions – often homozygous – and mutations, were shown to inactivate five negative regulators of the classical (*CYLD*) or alternative (*cIAP1&2, TRAF2, TRAF3,* and *NFKB2*) NFkB pathways [30]. Only two of these eleven genes – *NFKB2* and *TRAF3* – were found to have similar mutations or to be inactivated in both MM and B-cell tumors (Fig. 1A and above). In addition, different kinds of *TRAF2* abnormalities have been found in MM versus B-cell tumors. Some MM tumors and MMCL have homozygous deletion of *TRAF2*, which results in increased levels of *NIK*, the critical kinase that activates the alternative NFkB pathway. By contrast, Compagno [54] described missense mutations of *TRAF2* that were associated with enhanced activation of the classical NFkB pathway in B-cell lymphoma. These results are in accord with the idea that TRAF2 has two different functions – activation of the classical NFkB pathway (Fig. 1A) and inactivation of the alternative NFkB pathway (Fig. 1B). One possible explanation for the different pattern of mutations in MM and B-cell tumors is the absence in PC and MM cells of a functional BCR that is a key target for NFkB pathway mutations in B-cell tumors.

Based on studies with MMCLs, some of the NFkB mutations in MM tumors and MMCLs activate mainly the classical pathway (*CYLD, NFKB1, TACI*), and one mainly the alternative pathway (*NFKB2*), but most activate the alternative and to a lesser extent the classical pathway (*cIAP1/2, NIK, TRAF2, TRAF3, CD40*) (Fig.2B). All abnormalities in this last group result in increased levels of NIK protein, e.g., cIAP1/2, TRAF3 and TRAF2 are involved in a complex that negatively regulates NIK protein stability. This latter group of mutations are overrepresented, a result that is determined by the substantial prevalence of *TRAF3* mutations in both MMCLs (one third of mutations) and MM tumors (>50% of mutations) [29, 30, 62]. It is unclear why *TRAF3* mutations are predominant, but might be explained – at least in part by the presence of *TRAF3* on chr14, one of the chromosomes lost most frequently in MM, and particularly in non-hyperdiploid tumors [6]. By contrast, TRAF2 is located on chromosome 9, which is usually trisomic in hyperdiploid tumors, and only infrequently become monosomic. In addition, inactivation of TRAF2 might decrease activation of the classical pathway.

Previously, several groups have shown that regulation of NIK protein levels is mediated by a TRAF2/TRAF3 interaction, which recruits a TRAF2-cIAP1/2 ubiquitin ligase complex to a TRAF3-NIK complex and leads to the cIAP1/2mediated K48 ubiquitination of NIK that marks it for rapid proteasomal degradation [26, 27]. The activation of some TNFR (e.g., CD40 and BAFFR) results in an increased cIAP1/2-mediated K48 ubiquitination of TRAF3 that marks it for rapid proteasomal degradation, resulting in stabilization and activation of NIK that is not recruited to the TRAF2-cIAP1/2 complex. Mutations that inactivate TRAF3, TRAF2 and cIAP1/2 result in stabilization and activation of NIK, and consequent NFKB2 processing. The level of NIK protein is much higher in MMCLs that have inactivated either TRAF2 or cIAP1/2 than in MMCLs that have inactivated TRAF3. We showed that TRAF3 is not absolutely required for binding of TRAF2-cIAP1/2 to NIK in MMCL, even though the cIAP1/2-mediated degradation of NIK is substantially decreased in the absence of TRAF3. We also demonstrated the binding of TRAF2 to NIK in TRAF3 null MMCL, in contrast to the inability of others to identify an interaction of TRAF2 and NIK in other kinds of cells. It remains to be determined if some other protein is required for interaction of TRAF2 with NIK in TRAF3 deficient MMCLs. One possibility could be TNAP, a protein that can physically interact with NIK, TRAF2, and TRAF3, and that can suppress NIK kinase activity [63].

As indicated above high levels of NIK protein are able to activate not only the alternative pathway, but also the classical pathway in most MM cell lines. Others have shown that physiological or pathological increases in NIK expression in different type of cells can activate both pathways by a NIK and IKKα dependent mechanism [64]. However, it is not clear why there is a more severe defect in immune function in mice defective for NIK compared to mice that homozygously express kinase defective IKKα, and an even lesser effect in mice that are null for NFKB2 [65]. It also is surprising that a substantial IKKα shRNA knockdown has no apparent effect on NFkB activity of either pathway unless the residual levels of IKKα are sufficient for the NFkB activity observed in the MMCL [29, 66].

Selective activation or inactivation of either the classical or the alternative NFkB pathway in many MMCLs, respectively, increases or decreases the expression of all eleven genes that were used to generate an 11-gene NFkB index [29]. In addition, the long term effects of selective activation of either the alternative or the classical NFkB pathway demonstrated virtual identical changes in the gene expression pattern and gene set enrichment analysis (GSEA) on KEGG pathways in one MMCL, and similar albeit non-identical changes in a second MMCL [30]. The strong overlap of expression changes for activation of either NFkB pathway in MMCLs may explain why either pathway can be selectively activated by mutations in MM tumors. By contrast, the preponderance of mutations that selectively activate the classical NFkB pathway in B-cell tumors might be due to different biological effects for activation of the classical vs alternative NFkB pathway in these tumors.

Since the prevalence of NFkB mutations seems to be substantially higher in MMCLs compared to MM tumors, it seems possible that NFkB mutations often represent relatively late progression events, a conclusion that is supported by the heterogeneity of these mutations in some MM tumors [62]. Therefore, although NFkB activation in MM tumors results mainly from extrinsic signaling (Fig. 2 and above), the mutations that occur during progression and that constitutively activate NFkB would be expected to decrease dependence of tumor cells on the BM microenvironment (Fig.3).

The main features of NFkB activation in MM cells appear to be a strong dependence on microenvironment signals and a similar response to activation of either NFkB pathway. Given the apparent importance of NFkB activation for the survival of the MM tumor cells, inhibition of NFkB may be an attractive therapeutic approach.

### NFkB inhibitors in MM therapy

The outcome of multiple myeloma has substantially improved over the past decade, mainly due to recently approved drugs, such as bortezomib, thalidomide and lenalidomide. Increasing knowledge of the important role of the NFkB pathway in MM suggests the potential for the development of novel drugs that target this pathway.

Proteasome inhibitors, such as bortezomib or NPI-0052, have excellent clinical activity in patients with multiple myeloma [67]. In MM, bortezomib modifies c-FLIP levels, stabilizes pro-apoptotic members of the BCL-2 family, increases caspase activity, and inhibits the two major pathways leading to NFkB activation [68, 69]. It was shown that patients having MM tumors with a strong NFkB signature or with low level of TRAF3 RNA have a better response to bortezomib [62, 70]. Moreover, MM cells with low levels of TRAF3 showed resistant to dexamethasone treatment [62]. This might be explained, at least partially, by the mechanism that controls NIK protein levels. Treatment of cells with dexamethasone induces expression of IkBα, which inhibits the classical pathway, and also cIAP, which might block activity of the alternative NFkB pathway. However, abnormalities of TRAF2, TRAF3 or cIAP1/2, and also the downstream mutation of NFKB2 might minimize the effect of increased cIAP1/2 and decrease the effectiveness of dexamethasone. Hence, dexamethasone for treatment MM tumors that have mutations in genes regulating the alternative NFkB pathway might not be favourable.

In principle, the NFkB pathway could be targeted in MM tumors by blocking extrinsic activation of the pathway mediated by factors produced in the BM microenvironment, or by directly targeting intrinsic components of this pathway. As described above, the sequestration of BAFF and APRIL ligands by TACI.Fc results in a substantial decrease in normal murine BM PC. More importantly, an initial clinical trial treating MM patients with Atacicept (TACI.Ig) showed that the treatment is well-tolerated and decreased the amount of tumor in some patients [35]. To augment the inhibition of extrinsic signaling, and especially for the significant fraction of tumors that have acquired mutations in the NFkB pathway, it would seem useful to simultaneously use agents that block intrinsic components of both the classical and alternative NFkB pathways.

A number of IAP antagonists that mimic the interaction of SMAC (natural mammalian IAP antagonist) with IAPs have been developed. It was demonstrated that SMAC mimetics can promote apoptosis of various cancer cells including MM [71-74]. At the same time, we described that deletion of cIAP1/ 2 leads to strong activation of the NFkB. It's possible that inhibition of XIAP in MM cells leads to induction of apoptosis, but inhibition of cIAP leads to blocking of apoptosis. In any case, at this time use of SMAC mimetics for treatment of MM seems of questionable value, at least without further studies.

In fact, several inhibitors of IKKβ kinase, a critical component of the classical NFkB pathway have been developed. It has been shown that some of the IKKβ kinase inhibitors efficiently prevented growth of myeloma cells and induced apoptosis (even in the presence of bone marrow mononuclear cells) through caspase activation [29, 66, 75]. Unfortunately, specific inhibitors of the alternative NFkB pathway don't exist at this time. In view of findings that a substantial shRNA knock-down of IKKα kinase has no effect on NFkB activity in MMCL, this may not be the best molecule to target. Instead, NIK inhibitors would seem to be a better target for drug development for the following reasons. First, NIK is the key regulator of the alternative pathway and is the ultimate target of most of the mutations affecting the NFkB pathway in MM. Second, a knock-down of NIK expression with anti-NIK shRNA can inhibit both the alternative and classical NFkB pathways when both are activated as a result of increased levels of NIK. Ultimately, as they become available, combinations of NIK inhibitors, IKKβ inhibitors, and Atacicept may provide an effective therapeutic cocktail to target MGUS and MM tumors

## Abbreviations used

MM: Multiple myeloma
MMCLs: MM cell lines
MGUS: monoclonal gammopathy of undetermined significance
PC: plasma cells
BMSC: bone marrow stromal cells
BAFF: B cell–activating factor
APRIL: A proliferation-inducing ligand
BCR: B cell receptor
TNFR: tumor necrosis factor receptor
NIK: NFkB-inducing kinase
GCB DLBCL: Germinal Center B cell-like Diffuse Large B Cell Lymphoma
PMBL DLBCL: Primary Mediastinal B cell Lymphoma DLBCL
ABC DLBCL: Activated B Cell-like DLBCL
HL: Hodgkin's lymphoma
MALT.L: mucosa-associated lymphoid tissue lymphoma
FL: follicular lymphoma
WM: Waldenstrom's Macroglobulinemia

## References

[1] Fonseca R, Bergsagel PL, Drach J, Shaughnessy J, Gutierrez N, Stewart AK (2009). International Myeloma Working Group molecular classification of multiple myeloma: spotlight review. Leukemia.

[2] Fonseca R, Blood E, Rue M, Harrington D, Oken MM, Kyle RA (2003). Clinical and biologic implications of recurrent genomic aberrations in myeloma. Blood.

[3] Bergsagel PL, Kuehl WM, Zhan F, Sawyer J, Barlogie B, Shaughnessy J (2005). Cyclin D dysregulation: an early and unifying pathogenic event in multiple myeloma. Blood.

[4] Zhan F, Huang Y, Colla S, Stewart JP, Hanamura I, Gupta S (2006). The molecular classification of multiple myeloma. Blood.

[5] Fonseca R, Debes-Marun CS, Picken EB, Dewald GW, Bryant SC, Winkler JM (2003). The recurrent IgH translocations are highly associated with nonhyperdiploid variant multiple myeloma. Blood.

[6] Debes-Marun CS, Dewald GW, Bryant S, Picken E, SantanaDavila R, Gonzalez-Paz N (2003). Chromosome abnormalities clustering and its implications for pathogenesis and prognosis in myeloma. Leukemia.

[7] Zojer N, Konigsberg R, Ackermann J, Fritz E, Dallinger S, Kromer E (2000). Deletion of 13q14 remains an independent adverse prognostic variable in multiple myeloma despite its frequent detection by interphase fluorescence in situ hybridization. Blood.

[8] Dewald GW, Therneau T, Larson D, Lee YK, Fink S, Smoley S (2005). Relationship of patient survival and chromosome anomalies detected in metaphase and/or interphase cells at diagnosis of myeloma. Blood.

[9] Drach J, Ackermann J, Fritz E, Kromer E, Schuster R, Gisslinger H (1998). Presence of a p53 gene deletion in patients with multiple myeloma predicts for short survival after conventional-dose chemotherapy. Blood.

[10] Hanamura I, Stewart JP, Huang Y, Zhan F, Santra M, Sawyer JR (2006). Frequent gain of chromosome band 1q21 in plasmacell dyscrasias detected by fluorescence in situ hybridization: incidence increases from MGUS to relapsed myeloma and is related to prognosis and disease progression following tandem stem-cell transplantation. Blood.

[11] Caers J, Van Valckenborgh E, Menu E, Van Camp B, Vanderkerken K (2008). Unraveling the biology of multiple myeloma disease: cancer stem cells, acquired intracellular changes and interactions with the surrounding micro-environment. Bull Cancer.

[12] Lauta VM (2003). A review of the cytokine network in multiple myeloma: diagnostic, prognostic, and therapeutic implications. Cancer.

[13] Roodman GD (2004). Mechanisms of bone metastasis. N Engl J Med.

[14] Epstein J, Walker R (2006). Myeloma and bone disease: “the dangerous tango”. Clin Adv Hematol Oncol.

[15] Giuliani N, Rizzoli V, Roodman GD (2006). Multiple myeloma bone disease: Pathophysiology of osteoblast inhibition. Blood.

[16] Mitsiades CS, Mitsiades N, Poulaki V, Schlossman R, Akiyama M, Chauhan D (2002). Activation of NF-kappaB and upregulation of intracellular anti-apoptotic proteins via the IGF-1/Akt signaling in human multiple myeloma cells: therapeutic implications. Oncogene.

[17] Moreaux J, Veyrune JL, De Vos J, Klein B (2009). APRIL is overexpressed in cancer: link with tumor progression. BMC Cancer.

[18] Neri P, Kumar S, Fulciniti MT, Vallet S, Chhetri S, Mukherjee S (2007). Neutralizing B-cell activating factor antibody improves survival and inhibits osteoclastogenesis in a severe combined immunodeficient human multiple myeloma model. Clin Cancer Res.

[19] Ghosh S, Karin M (2002). Missing pieces in the NF-kappaB puzzle. Cell.

[20] Hayden MS, Ghosh S (2008). Shared principles in NF-kappaB signaling. Cell.

[21] Dejardin E (2006). The alternative NF-kappaB pathway from biochemistry to biology: pitfalls and promises for future drug development. Biochem Pharmacol.

[22] Claudio E, Brown K, Park S, Wang H, Siebenlist U (2002). BAFFinduced NEMO-independent processing of NF-kappa B2 in maturing B cells. Nat Immunol.

[23] Coope HJ, Atkinson PG, Huhse B, Belich M, Janzen J, Holman MJ (2002). CD40 regulates the processing of NF-kappaB2 p100 to p52. EMBO J.

[24] Vallabhapurapu S, Karin M (2009). Regulation and function of NFkappaB transcription factors in the immune system. Annu Rev Immunol.

[25] Liao G, Zhang M, Harhaj EW, Sun SC (2004). Regulation of the NF-kappaB-inducing kinase by tumor necrosis factor receptor-associated factor 3-induced degradation. J Biol Chem.

[26] Zarnegar BJ, Wang Y, Mahoney DJ, Dempsey PW, Cheung HH, He J (2008). Noncanonical NF-kappaB activation requires coordinated assembly of a regulatory complex of the adaptors cIAP1, cIAP2, TRAF2 and TRAF3 and the kinase NIK. Nat Immunol.

[27] Vallabhapurapu S, Matsuzawa A, Zhang W, Tseng PH, Keats JJ, Wang H (2008). Nonredundant and complementary functions of TRAF2 and TRAF3 in a ubiquitination cascade that activates NIK-dependent alternative NF-kappaB signaling. Nat Immunol.

[28] Varfolomeev E, Goncharov T, Fedorova AV, Dynek JN, Zobel K, Deshayes K (2008). c-IAP1 and c-IAP2 are critical mediators of tumor necrosis factor alpha (TNFalpha)-induced NFkappaB activation. J Biol Chem.

[29] Annunziata CM, Davis RE, Demchenko Y, Bellamy W, Gabrea A, Zhan F (2007). Frequent engagement of the classical and alternative NF-kappaB pathways by diverse genetic abnormalities in multiple myeloma. Cancer Cell.

[30] Demchenko YN, Glebov OK, Zingone A, Keats JJ, Bergsagel PL, Kuehl WM (2010). Classical and/or alternative NF{kappa}B pathway activation in multiple myeloma. Blood.

[31] O'Connor BP, Raman VS, Erickson LD, Cook WJ, Weaver LK, Ahonen C (2004). BCMA is essential for the survival of long-lived bone marrow plasma cells. J Exp Med.

[32] Moreaux J, Legouffe E, Jourdan E, Quittet P, Reme T, Lugagne C (2004). BAFF and APRIL protect myeloma cells from apoptosis induced by interleukin 6 deprivation and dexamethasone. Blood.

[33] Abe M, Kido S, Hiasa M, Nakano A, Oda A, Amou H (2006). BAFF and APRIL as osteoclast-derived survival factors for myeloma cells: a rationale for TACI-Fc treatment in patients with multiple myeloma. Leukemia.

[34] Yaccoby S, Pennisi A, Li X, Dillon SR, Zhan F, Barlogie B (2008). Atacicept (TACI-Ig) inhibits growth of TACI(high) primary myeloma cells in SCID-hu mice and in coculture with osteoclasts. Leukemia.

[35] Rossi JF, Moreaux J, Hose D, Requirand G, Rose M, Rouille V (2009). Atacicept in relapsed/refractory multiple myeloma or active Waldenstrom's macroglobulinemia: a phase I study. Br J Cancer.

[36] Davis RE, Brown KD, Siebenlist U, Staudt LM (2001). Constitutive nuclear factor kappaB activity is required for survival of activated B cell-like diffuse large B cell lymphoma cells. J Exp Med.

[37] Rosenwald A, Wright G, Leroy K, Yu X, Gaulard P, Gascoyne RD (2003). Molecular diagnosis of primary mediastinal B cell lymphoma identifies a clinically favorable subgroup of diffuse large B cell lymphoma related to Hodgkin lymphoma. J Exp Med.

[38] Staudt LM, Dave S (2005). The biology of human lymphoid malignancies revealed by gene expression profiling. Adv Immunol.

[39] Packham G (2008). The role of NF-kappaB in lymphoid malignancies. Br J Haematol.

[40] Re D, Hartlapp I, Greiner A, Diehl V, Wickenhauser C (2008). Analysis of CARMA1/BCL10/MALT1 expression in Reed-Sternberg cells of classical Hodgkin lymphoma. Leuk Lymphoma.

[41] Lucas PC, Yonezumi M, Inohara N, McAllister-Lucas LM, Abazeed ME, Chen FF (2001). Bcl10 and MALT1, independent targets of chromosomal translocation in malt lymphoma, cooperate in a novel NF-kappa B signaling pathway. J Biol Chem.

[42] Che T, You Y, Wang D, Tanner MJ, Dixit VM, Lin X (2004). MALT1/paracaspase is a signaling component downstream of CARMA1 and mediates T cell receptor-induced NF-kappaB activation. J Biol Chem.

[43] Sun L, Deng L, Ea CK, Xia ZP, Chen ZJ (2004). The TRAF6 ubiquitin ligase and TAK1 kinase mediate IKK activation by BCL10 and MALT1 in T lymphocytes. Mol Cell.

[44] Coornaert B, Baens M, Heyninck K, Bekaert T, Haegman M, Staal J (2008). T cell antigen receptor stimulation induces MALT1 paracaspase-mediated cleavage of the NF-kappaB inhibitor A20. Nat Immunol.

[45] Du MQ (2007). MALT lymphoma : recent advances in aetiology and molecular genetics. J Clin Exp Hematop.

[46] Liu H, Ye H, Dogan A, Ranaldi R, Hamoudi RA, Bearzi I (2001). T(11;18)(q21;q21) is associated with advanced mucosaassociated lymphoid tissue lymphoma that expresses nuclear BCL10. Blood.

[47] Levy M, Copie-Bergman C, Gameiro C, Chaumette MT, Delfau-Larue MH, Haioun C (2005). Prognostic value of translocation t(11;18) in tumoral response of low-grade gastric lymphoma of mucosa-associated lymphoid tissue type to oral chemotherapy. J Clin Oncol.

[48] Kingeter LM, Schaefer BC (2010). Malt1 and cIAP2-Malt1 as effectors of NF-kappaB activation: kissing cousins or distant relatives?. Cell Signal.

[49] Ngo VN, Davis RE, Lamy L, Yu X, Zhao H, Lenz G (2006). A loss-of-function RNA interference screen for molecular targets in cancer. Nature.

[50] Davis RE, Ngo VN, Lenz G, Tolar P, Young RM, Romesser PB (2010). Chronic active B-cell-receptor signalling in diffuse large B-cell lymphoma. Nature.

[51] Schmitz R, Hansmann ML, Bohle V, Martin-Subero JI, Hartmann S, Mechtersheimer G (2009). TNFAIP3 (A20) is a tumor suppressor gene in Hodgkin lymphoma and primary mediastinal B cell lymphoma. J Exp Med.

[52] Kato M, Sanada M, Kato I, Sato Y, Takita J, Takeuchi K (2009). Frequent inactivation of A20 in B-cell lymphomas. Nature.

[53] Braggio E, Keats JJ, Leleu X, Van Wier S, Jimenez-Zepeda VH, Valdez R (2009). Identification of copy number abnormalities and inactivating mutations in two negative regulators of nuclear factor-kappaB signaling pathways in Waldenstrom's macroglobulinemia. Cancer Res.

[54] Compagno M, Lim WK, Grunn A, Nandula SV, Brahmachary M, Shen Q (2009). Mutations of multiple genes cause deregulation of NF-kappaB in diffuse large B-cell lymphoma. Nature.

[55] Cabannes E, Khan G, Aillet F, Jarrett RF, Hay RT (1999). Mutations in the IkBa gene in Hodgkin's disease suggest a tumour suppressor role for IkappaBalpha. Oncogene.

[56] Martin-Subero JI, Gesk S, Harder L, Sonoki T, Tucker PW, Schlegelberger B (2002). Recurrent involvement of the REL and BCL11A loci in classical Hodgkin lymphoma. Blood.

[57] Courtois G, Gilmore TD (2006). Mutations in the NF-kappaB signaling pathway: implications for human disease. Oncogene.

[58] Feuerhake F, Kutok JL, Monti S, Chen W, LaCasce AS, Cattoretti G (2005). NFkappaB activity, function, and targetgene signatures in primary mediastinal large B-cell lymphoma and diffuse large B-cell lymphoma subtypes. Blood.

[59] Fracchiolla NS, Lombardi L, Salina M, Migliazza A, Baldini L, Berti E (1993). Structural alterations of the NF-kappa B transcription factor lyt-10 in lymphoid malignancies. Oncogene.

[60] Rayet B, Gelinas C (1999). Aberrant rel/nfkb genes and activity in human cancer. Oncogene.

[61] Nagel I, Bug S, Tonnies H, Ammerpohl O, Richter J, Vater I (2009). Biallelic inactivation of TRAF3 in a subset of B-cell lymphomas with interstitial del(14)(q24.1q32.33). Leukemia.

[62] Keats JJ, Fonseca R, Chesi M, Schop R, Baker A, Chng WJ (2007). Promiscuous mutations activate the noncanonical NF-kappaB pathway in multiple myeloma. Cancer Cell.

[63] Hu WH, Mo XM, Walters WM, Brambilla R, Bethea JR (2004). TNAP, a novel repressor of NF-kappaB-inducing kinase, suppresses NF-kappaB activation. J Biol Chem.

[64] Zarnegar B, Yamazaki S, He JQ, Cheng G (2008). Control of canonical NF-kappaB activation through the NIK-IKK complex pathway. Proc Natl Acad Sci U S A.

[65] Mills DM, Bonizzi G, Karin M, Rickert RC (2007). Regulation of late B cell differentiation by intrinsic IKKalpha-dependent signals. Proc Natl Acad Sci U S A.

[66] Hideshima T, Chauhan D, Kiziltepe T, Ikeda H, Okawa Y, Podar K (2009). Biologic sequelae of I{kappa}B kinase (IKK) inhibition in multiple myeloma: therapeutic implications. Blood.

[67] McConkey DJ, Zhu K (2008). Mechanisms of proteasome inhibitor action and resistance in cancer. Drug Resist Updat.

[68] Mitsiades N, Mitsiades CS, Poulaki V, Chauhan D, Fanourakis G, Gu X (2002). Molecular sequelae of proteasome inhibition in human multiple myeloma cells. Proc Natl Acad Sci U S A.

[69] Hideshima T, Chauhan D, Hayashi T, Akiyama M, Mitsiades N, Mitsiades C (2003). Proteasome inhibitor PS-341 abrogates IL-6 triggered signaling cascades via caspase-dependent downregulation of gp130 in multiple myeloma. Oncogene.

[70] Chng WJ, Kumar S, Vanwier S, Ahmann G, Price-Troska T, Henderson K (2007). Molecular dissection of hyperdiploid multiple myeloma by gene expression profiling. Cancer Res.

[71] Petersen SL, Wang L, Yalcin-Chin A, Li L, Peyton M, Minna J (2007). Autocrine TNFalpha signaling renders human cancer cells susceptible to Smac-mimetic-induced apoptosis. Cancer Cell.

[72] Baud V, Karin M (2009). Is NF-kappaB a good target for cancer therapy? Hopes and pitfalls. Nat Rev Drug Discov.

[73] Chauhan D, Neri P, Velankar M, Podar K, Hideshima T, Fulciniti M (2007). Targeting mitochondrial factor Smac/DIABLO as therapy for multiple myeloma (MM). Blood.

[74] Desplanques G, Giuliani N, Delsignore R, Rizzoli V, Bataille R, Barille-Nion S (2009). Impact of XIAP protein levels on the survival of myeloma cells. Haematologica.

[75] Romagnoli M, Desplanques G, Maiga S, Legouill S, Dreano M, Bataille R (2007). Canonical nuclear factor kappaB pathway inhibition blocks myeloma cell growth and induces apoptosis in strong synergy with TRAIL. Clin Cancer Res.

